# Polycyclic Aromatic Hydrocarbons in Indoor Dust in Croatia: Levels, Sources, and Human Health Risks

**DOI:** 10.3390/ijerph191911848

**Published:** 2022-09-20

**Authors:** Ivana Jakovljević, Marija Dvoršćak, Karla Jagić, Darija Klinčić

**Affiliations:** 1 Environmental Hygiene Unit, Institute for Medical Research and Occupational Health, 10000 Zagreb, Croatia; 2 Biochemistry and Organic Analytical Chemistry Unit, Institute for Medical Research and Occupational Health, 10000 Zagreb, Croatia

**Keywords:** PAHs, house dust, HPLC, carcinogenic risk assessment, ILCR

## Abstract

Compounds that contribute to indoor pollution are regularly investigated due to the fact that people spend most of their time indoors. Worldwide investigations have shown that polycyclic aromatic hydrocarbons (PAHs) are present in indoor dust, but to the best of our knowledge, this paper reports for the first time the presence of PAHs in Croatian households. Eleven PAHs were analysed in house dust samples collected in the city of Zagreb and surroundings (N = 66). Their possible indoor sources and the associated health risks were assessed. Total mass fraction of detected PAHs ranged from 92.9 ng g^−1^ to 1504.1 ng g^−1^ (median 466.8 ng g^−1^), whereby four-ring compounds, Flu and Pyr, contributed the most. DahA was the only compound that did not show statistically significantly positive correlation with other analysed PAHs, indicating that it originated from different sources. Based on diagnostic ratios and principal component analysis (PCA), mixed sources contributed to PAHs levels present in Croatian households. Although our results indicate that Croatian house dusts are weakly polluted with PAHs, total ILCR values calculated for children and adults revealed that people exposed to the highest mass fractions of PAHs measured in this area are at elevated cancer risk.

## 1. Introduction

Since people nowadays spend most of their time indoors, pollutants present in the interior have a significant impact on human health. Modern indoor environments contain a number of potential sources of indoor pollution, such as combustion products of cooking, heating, and smoking, building materials, consumer products, and radiation. A significant proportion of indoor contaminants can be adsorbed to suspended indoor air particles or to house dust particles that are ubiquitously deposited [1]. House dust is a complex mixture of different biologically derived material and particles of both, indoor and outdoor origin. Which components will be dominant in house dust depends on numerous factors, such as environmental and seasonal factors, air filtration, and homeowner activities [1]. House dust particles and compounds adsorbed to house dust may enter the human body by dermal adsorption, nondietary ingestion, and inhalation. The contribution of inhalation to total exposure appears to be considerably lower compared to the other two pathways, while the ingestion of house dust is of specific concern for children who often put their hands or toys into their mouths. Because of all this, house dust has been recognized as a significant source of indoor exposure to hazardous substances, such as polychlorinated biphenyls, different classes of flame retardants, metals [2,3,4,5,6,7], and also polycyclic aromatic hydrocarbons (PAHs) that are ubiquitous in indoor and ambient environments [8,9,10]. These compounds are also classified as possibly or probably mutagenic and carcinogenic to humans [11]. PAHs mainly originate from anthropogenic sources and are formed as products of incomplete combustion during industrial activities or engine fuels, as products of cooking, by-products of house heating, cigarette smoke, candle burning, and the intentional or unintentional burning of wood [7,12,13,14,15,16,17]. Although PAHs are mostly generated outdoors, numerous studies have shown that they contribute to a great extent to indoor pollution [9,18,19,20]. Given that today most people spend up to 90% of their time indoors, such as at home, in the workplace, at school, etc., their impact on human health from indoor sources, especially through dust, is not negligible. Exposure to PAHs in different indoor environments around the world was evaluated, but a smaller part refers to the data from Europe [10,18,20,21,22,23], and as far as we know, there is no information on indoor PAH presence for Croatia.

Therefore, the main objectives of this study were: (i) to determine the levels of 11 PAHs in household dust from the city of Zagreb, Croatia for the first time; (ii) to explore possible sources of these contaminants on the basis of the relationship between their concentrations and house characteristics and the habits of residents; and (iii) to conduct a health risk assessment and to evaluate the cancer risks of exposure to PAHs in residential environments.

## 2. Materials and Methods

### 2.1. Collection of House Dust Samples

Collection of house dust samples is described in detail in the study of Klinčić et al. [24]. Briefly, house dust samples (N = 66) were collected between October 2020 and February 2021 in different households in Zagreb and surroundings. All study participants were invited to fill in a complete questionnaire. After removal of non-dust particles (e.g., plastics, hair, paper), each house dust sample was sieved twice (500 μm stainless steel mesh) and then homogenized by a rotary mixer for 24 h. All house dust samples were kept in a glass container at room temperature in a dark place.

### 2.2. PAH Analysis

Eleven PAHs were analysed in our investigation: fluoranthene (Flu), pyrene (Pyr), chrysene (Chry), benzo[*a*]anthracene (BaA), benzo[*j*]fluoranthene (BjF), benzo[*b*]fluoranthene (BbF), benzo[*a*]pyrene (BaP), benzo[*k*]fluoranthene (BkF), benzo[*ghi*]perylene (BghiP), dibenzo[a,h]anthracene (DahA), and indeno [1,2,3-*c,d*]pyrene (IP). The accelerated solvent extraction (ASE) was used to extract PAHs from house dust. Approximately 0.5 g of each dust sample was weighed; the samples were mixed with a roast sand and transferred in an extraction cell. The extraction was conducted at 125 °C in two static cycles, 5 min each. A mixture of cyclohexane and toluene (30:70, *v*/*v*) was used as extraction solvent. Extracts were collected in ASE vials (60 mL) and evaporated to dryness by Rocket GeneVac. After evaporation, samples were resolved in 1000 µL of acetonitrile, centrifuged 10 min at 3000 rpm, and passed through a syringe filter (PTFE) 13 mm, 0.2 µm into the vial. High performance liquid chromatography (HPLC) with a fluorescence detector was used to determine the mass fractions of PAHs in house dust. Data quality assurance and control were achieved by analyzing random samples before and after spiking with standard solution EPA 610 PAH mix. The average recoveries of all PAHs in spiked samples ranged between 80% (BaA) to 99% (Chry). The method detection limit (DL) ranged from 0.04 ng g^−1^ for Pyr to 2.18 ng g^−1^ for BghiP, while the DL for BaP was 1.2 ng g^−1^ [25,26]. Concentrations of PAHs were determined from a five point calibration curve ranging from 0.005 ng µL^−1^ to 0.08 ng µL^−1^ for Pyr, BaA, Chry, BkF, BaP, and IP, and from 0.01 ng µL^−1^ to 0.16 ng µL^−1^ for Flu, BjF, BbF, DahA, and BghiP.

### 2.3. Statistical Analysis

Microsoft Excel and the Statistica 13 (Tibco Software Inc.; Palo Alto, CA, USA) program were used. Statistical significance was set at 5% (*p* < 0.05). To assess the normality of the data, the Shapiro–Wilk test was used. The influence of the type of household, renovation activity, and occupant habits regarding indoor smoking on PAH concentrations were tested by *t*-test, and a logarithmic transformation was applied to normalize the distribution of PAH concentrations. Analysis of variance (one-way ANOVA post hoc test) was used to examine the association between house age, type of flooring, frequency of natural air ventilation, vacuum cleaning frequency, and type of house heating and PAH dust concentrations. Logarithmic transformation was used on PAH concentrations to approximate normal distribution. To estimate possible pollution sources, the concentration ratios between selected PAHs were calculated, and the multivariate principal component analysis (PCA) was performed. Principal components (PCs) with eigenvalues greater than 1 for samples was calculated, and their contributions to the total variance were determined. Additionally, stepwise multiple linear regression analysis for each individual PAH was used to identify which of the considered household variables (e.g., age of the house, number of residents, renovation activity…) were the best predictors of the PAH concentration. Prior to regression analysis, dependent variables (PAH concentration) were logarithmically transformed to approach normal distribution.

### 2.4. Human Exposure

The incremental lifetime cancer risk (ILCR) model was used in our study to estimate the probability of an individual developing any type of cancer from the lifetime exposure to a particular carcinogen. This model uses a toxic equivalent factor (TEF) approach based on the assignment of toxic potency to each PAH in the mixture relative to toxic potency of BaP which is considered as the reference compound due to its strong carcinogenic effect on humans [19,27,28,29]. Total carcinogenic potency (TCP) is the sum of BaP equivalents (BaP_eq_) which are multipliers of individual PAH mass fractions (γ_i_, ng g^−1^) in dust and its assigned TEFi (Equation (1)). In this study, TEFs set by Nisbet and LaGoy were used [28].

(1) $$\mathrm{TCP}=\sum {\mathrm{BaP}}_{\mathrm{eq}}=\sum \gamma_{i}\times {\mathrm{TEF}}_{i}$$

Human exposure to carcinogenic compounds in house dust can be through inhalation, dermal absorption, and dust ingestion. Since the contribution of inhalation to total exposure appears to be considerably lower than the contribution of ingestion and dermal absorption [19], only cancer risks via ingestion (Equation (2)) and dermal absorption (Equation (3)) of indoor dust as exposure routes were determined, and their sum was taken to evaluate total cancer risk (ILCR_tot_) of human exposure to PAHs during lifetime (Equation (4)). The values of parameters used for calculations are shown in Table S1 of the Supplementary Data section.

(2) $${\mathrm{ILCR}}_{\mathrm{ing}}={\mathrm{CSF}}_{\mathrm{ing}}\times \frac{\mathrm{TCP}\;\times {\mathrm{IR}}_{\mathrm{ing}}\times \mathrm{EF}\times \mathrm{ED}\times \mathrm{CF}}{\mathrm{BW}\times \mathrm{AT}}$$

(3) $${\mathrm{ILCR}}_{\mathrm{derm}}={\mathrm{CSF}}_{\mathrm{derm}}\times \frac{\mathrm{TCP}\;\times \mathrm{SA}\times \mathrm{AF}\times \mathrm{EF}\times \mathrm{ED}\times \mathrm{CF}\times \mathrm{ABS}}{\mathrm{BW}\times \mathrm{AT}}$$

The cancer slope factor (CSF) is defined as an upper-bound of the probability of a response per unit intake of a chemical over a lifetime according to the US EPA (1989) [29]. For BaP, CSF through ingestion and dermal absorption are 7.3 (mg kg^−1^ d^−1^)^−1^ and 25 (mg kg^−1^ d^−1^)^−1^, respectively [27]. IR_ing_ is the dust ingestion rate (mg day^−1^), EF is the exposure frequency (day year^−1^), ED is the exposure duration (day), CF is the conversion factor (10^−6^ kg mg^−1^), BW is body weight (kg), AT is average life span (day), SA is surface area of the skin exposed to dust (cm^2^), AF is the skin adherence factor (mg cm^−2^), and ABS is the dermal absorption factor (unitless). F is the fraction of time spent daily in each microenvironment. Time fractions spent at home were taken to be 86% for children and 64% for adults.

(4) $${\mathrm{ILCR}}_{\mathrm{tot}}={\mathrm{ILCR}}_{\mathrm{ing}}+{\mathrm{ILCR}}_{\mathrm{derm}}$$

According to the literature, cancer risk levels less than 10^−6^ are considered as negligible to human health, levels between 10^−6^ and 10^−4^ indicate potential carcinogenic effect of PAHs to humans, and if levels exceed 10^−4^, there may be indications of a high cancer risk due to human exposure to PAHs.

## 3. Results

### 3.1. Levels and Profile of PAHs in House Dust

Eleven selected PAHs were analyzed and detected in all dust samples (N = 66), except BjF, which was not detected in only one dust sample. A descriptive summary of PAH concentrations in house dust samples is provided in Table 1. The sum of the measured mass fractions of PAHs (ΣPAHs) ranged from 92.9 ng g^−1^ to 1504.1 ng g^−1^, with a median of 466.8 ng g^−1^. Flu and Pyr contributed the most to the total sum of measured PAH mass fractions with medians of 25% and 22%, respectively. Dominance of the PAHs with four rings in Croatian households is in accordance with the literature data [2,20,30]. Previous investigations of PAHs in the ambient air in the Zagreb city area suggested that higher mass concentrations of those higher molecular weight PAHs were detected in air particulate matter, especially during the heating season [25,31]. Since the dust samples analyzed in this study were collected during fall and winter, their input from outdoors could be the reason for the detected higher concentrations.

BaP is designated by the International Agency for Research on Cancer as a group 1 (known human carcinogen), and it is used as an indicator of carcinogenic hazard in different polluted environments. BaP is the most commonly measured PAH and usually taken as the representative of the whole PAH group [32,33,34]. Mass fractions of BaP in house dust higher than 10 µg g^−1^ are stated by the German Federal Environmental Agency’s Commission for Indoor Air Quality to have adverse human health effects [8]. Mass concentration of BaP in Croatian house dusts ranged from 2.9 ng g^−1^ to 106 ng g^−1^, with a median of 17.24 ng g^−1^; therefore, they were three orders of magnitude lower than the established limit value. Among several PAHs suspected to be carcinogenic, BaA is the most prominent, and in our study mass fractions of BaA, were similar to those of BaP. Total mass fractions of most carcinogenic PAHs (BaP, BaA, BbF, BkF, DahA, and IP) in Croatian house dusts ranged from 23.98 ng^−1^ to 512.8 ng g^−1^, while less carcinogenic PAHs were about two times higher and ranged from 68.9 ng g^−1^ to 1096.7 ng g^−1^.

Since there are no relevant criteria for assessing the permissible and tolerable levels of PAHs in indoor dusts, some authors [7,20] used soil criteria proposed by Maliszewska-Kordybach [35] in order to classify the level of house dust pollution by PAHs. According to this criteria, samples with ∑PAHs > 1000 ng g^−1^ are considered as heavily polluted, 600 ng g^−1^ > ∑PAHs > 1000 ng g^−1^ polluted, 200 ng g^−1^ > ∑PAHs > 600 ng g^−1^ weakly polluted, and samples with ∑PAHs < 200 ng g^−1^ are considered as not polluted. Our results suggest that the majority of samples collected in the city of Zagreb are weakly polluted with PAHs (56% of analyzed samples), while 27% of the analyzed samples might be considered as polluted, 11% as not polluted, and only 6% as heavily polluted. Such results indicate that the household dust samples from Croatia are less polluted by PAHs in comparison with the PAH dust levels measured in other residential environments around the world (Table 2). Similar concentrations were reported for house dusts from Greece sampled a decade ago [21] or slightly higher in house dusts from Nigeria [14], while the lower concentrations were reported only for house dust from the Czech Republic [23]. By far the highest PAH levels in indoor dust samples were measured in Canada [8], China [36], Saudi Arabia [15], and Ecuadorian Amazonia [7], but PAH levels higher than in Croatian households were also reported in recent research from Greece [10,22] and in research from our neighboring countries, Italy [18] and Serbia [20] (Table 2). Nevertheless, caution is needed when comparing results obtained in different studies, and multiple factors should be considered, such as the number of analyzed dust samples, number of analyzed PAHs, vicinity of potential PAH sources in the sampling location, type of heating, habits of residents, etc.

### 3.2. Correlation between PAH Mass Fractions and Household Factors

No significant differences were found between house renovation activity and PAH mass fractions, while results of the *t*-test indicate that higher mass fractions of BghiP and IP are obtained in houses than in apartments. Those two compounds, along with BaP, are indicative of vehicular traffic emissions [14,40] according to some authors. DahA was the only PAH for which smoking was found to be a positive predictor. There are several studies which associate elevated concentrations of PAHs in house dust with in-house smoking [4,8,10,17,18,22]; however, data on the exact PAH compounds of which concentrations are affected by smoking are not uniform. Therefore, few authors agree that the effect of smoking on PAH concentrations in dust is still uncertain [22,30]. The absence of the correlations of concentrations of other PAHs with smoking in the present study may be a result of a relatively small number of households that reported in-house smoking.

According to the data from questionnaires, four types of house heating were used in sampling households—electricity, gas heating, wood burning, and heating plant. Results of the one-way ANOVA test revealed statistically significantly higher mass fractions of Chry, Bbf, BkF, BaP, IP, and ∑PAHs in dust from households that used wood burning for heating and households heated by the heating plants. Similar statistics were reported in the study of Mannino and Orecchio [18] in dust samples collected in houses heated by a fireplace. These results are not surprising, because wood burning, especially its incomplete combustion is an important source of PAHs [42,43,44]. Although some authors have found that wood burning results in higher emissions of Flu and Pyr into the air [42,44], it has to be considered that these PAHs are more volatile than others and probably evaporate from household dust over time. Our results are also in accordance with the literature data reported by Živančev et al. [20], Al-Harbi et al. [2], and Qi et al. [30]. Furthermore, due to the fact that dust samples were collected in fall and winter (heating season), PAH input from outdoors could also be the reason for their higher concentrations in dust. Previous investigations of PAHs in the ambient air in the Zagreb city area [25,31] reported higher mass concentrations of PAHs in ambient particulate matter during the heating season and identified wood burning as one of their sources. In this study, a statistically significant difference was also obtained in the mass fraction of BbF detected in households heated by electricity and households heated by the heating plants, while the age of the house, the type of flooring, ventilation frequency, and vacuum cleaning frequency did not affect measured PAH mass fractions.

Spearman’s rank correlation coefficients (Table 3) revealed significant positive correlations at a high level of confidence (*p* < 0.001) between all measured PAHs (r ranged between 0.389 and 0.956) with exception of DahA. Such results suggest that similar factors, possibly common sources, influence the observed mass fractions of all PAHs, except DahA, which is in accordance with the previous statement that only DahA correlates with residents’ smoking habits. In order to obtain a better insight into the potential influences on indoor PAH mass fractions, Spearman’s rank correlation between the measured PAH mass fractions and different household factors was conducted, and results are summarized in Table 4. Statistically significant correlations were observed for a small number of combinations. Results indicated that BjF mass fraction correlated statistically significantly with the largest number of the observed household factors, namely with the house square footage (m^2^), carpeted area (m^2^), number of curtains, number of electronic devices, and the number of hours they were in use. The number of curtains and time spent using electronic devices (hours per day) were two household factors that correlated statistically significantly with the highest number of PAHs, and they are, in addition to the number of electronic devices, factors that positively (not necessarily statistically significantly) correlated with the mass fractions of all measured PAHs. Generally, those are the factors known to contribute to the house dust formation and/or serve as a reservoir for it. Ren et al. [45] reported that some PAHs can be deposited in indoor dust following emission from the heated plastic material in running computers. Furthermore, in a mentioned study, among the most abundant PAH compounds were three (Chry, BbF, and BaP) out of four PAHs that in our study showed significantly positive correlation with time spent using electronic devices. Our results indicated that house age correlated positively to the ten out of the eleven PAHs (statistically significant only for BkF). Negative correlations, but not statistically significant, were observed between the mass fraction of the majority of PAHs with the number of residents and the number of upholstered pieces of furniture.

### 3.3. Identification of PAHs Sources

**Figure 1:**
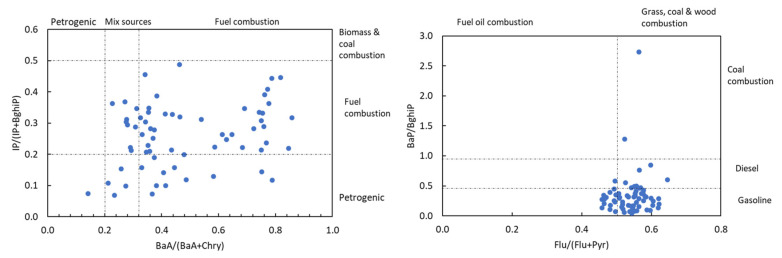
PAH diagnostic ratios for the identification of pollution emission sources in household dust samples from the city of Zagreb.

The diagnostic ratios of PAHs are often used as tools for identifying their potential sources. Generally, PAHs are formed as products of different combustion processes, and their sources can be divided into two groups, petrogenic and pyrogenic. Petrogenic sources usually include combustion of petroleum products, and mainly lower molecular weight PAHs are produced (two or three aromatic rings), while from pyrogenic sources which include diesel and gasoline combustion, combustion of natural gas, coal, and other fossil fuels, mainly higher molecular PAHs are produced (four or more aromatic rings) [38]. Several diagnostic ratios were applied to identify the sources of PAHs present in house dust from the city of Zagreb (Figure 1). Results showed that Flu(Flu + Pyr) was in a range from 0.46 to 0.65 (average 0.54), suggesting PAHs came from mixed sources of traffic emissions and industrial combustion processes, while BaP/BghiP ratios ranged from 0.14 to 0.86 (average 0.49), suggesting vehicular traffic as the dominant source of PAHs. The IP(IP + BghiP) ratios of household dusts in our investigation were from 0.07 to 0.49 (average 0.26), suggesting mixed PAH sources (fuel combustion and petrogenic), which are consistent with the implications obtained based on the ratios of Flu/(Flu + Pyr). Ratios of BaP/BghiP ranged from 0.04 to 2.73 (average 0.34), suggesting that PAHs were generated from combustion of a gasoline fuel.

**Figure 2:**
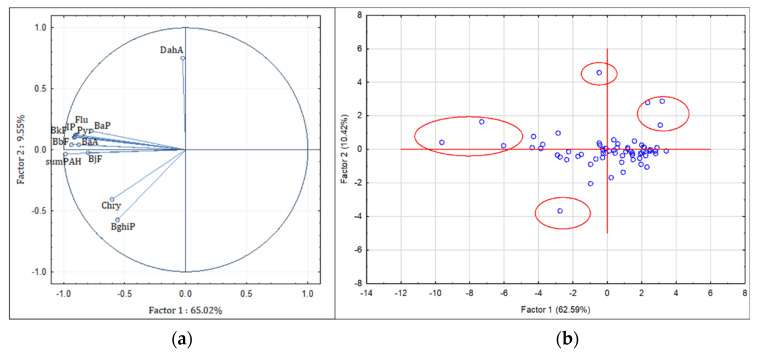
Loading/variable plot of first two principal components for PAHs (**a**) and score plot obtained for ∑PAHs in individual sampling location (**b**) measured in household dust samples from the city of Zagreb.

For obtaining a more complete PAHs source apportionment analysis, principal component analysis (PCA) was performed. Figure 2a shows the loadings/variable plot constructed using the first two principal components allowing for a visualization of parameter grouping. The factor analysis confirmed the relationship among the measured PAHs in house dust samples from the city of Zagreb and two extracted factors (F1—65.02% and F2—9.55%) accounting for 74.57% of the total variance. Factor 1 was highly loaded with BaP, BaA, Pyr, Flu, BkF, IP, BbF, BjF, and ∑PAHs. Even though Spearman correlation coefficients were strong between any two of the PAH compounds except DahA, those results indicate that the sources of grouped congeners potentially differ from the sources of Chry and BghiP. Factor 2 contained a positive loading of DahA, that previous results indicated had weaker Spearman correlation coefficients with other investigated PAHs, and whose concentrations were correlated with smoking inside the households. The sample sites on the PCA score plot are shown in Figure 2b, with most of the sites located near the origin which represents the mean concentration of all samples. It can be observed that some samples were excluded as outliers, but there are no uniform common factors between them. Samples that are singled out on the left side of the PCA score plot are four samples that are characterized as heavily polluted samples with ∑PAH > 1000 ng g^−1^. Residents of these households reported longer times of active usage of electronic devices than the average reported in by others, wood heating was used in one of them, and in-house smoking was not reported in any of them. Unlike that, smokers were reported in three out of four households singled out on the upper right part of the PCA score plot. Such results confirm that PAH concentrations in indoor dust samples are influenced by a number of indoor and outdoor factors. Nevertheless, results of PCA analysis were in very good agreement with results of the diagnostic ratios, both clearly indicating mixed sources of PAHs in house dust.

### 3.4. Human Exposure Assessment

**Figure 3:**
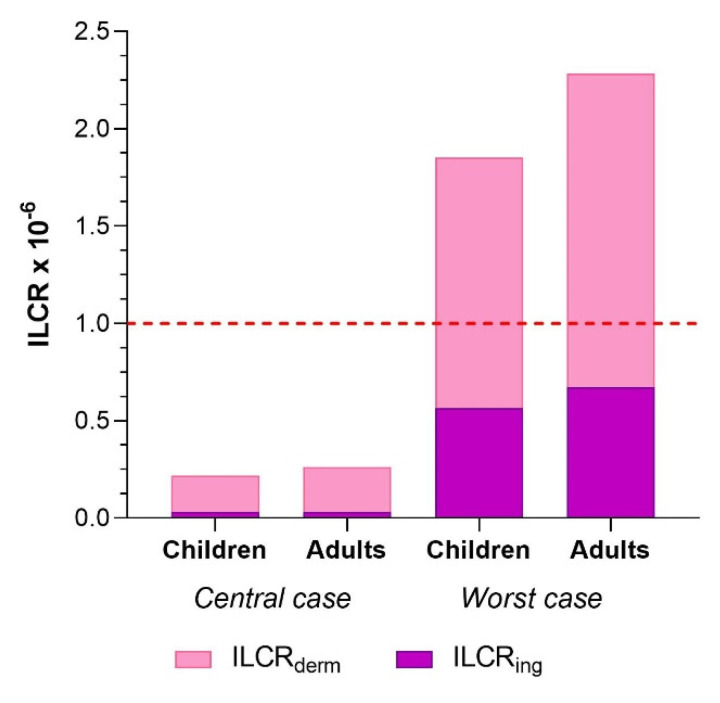
Incremental lifetime cancer risk (ILCRs) for children and adults from PAHs in household dust samples from the city of Zagreb.

The ILCR was calculated to quantify the potential cancer risk for the Croatian citizens of two different age groups, children (1–6 years old) and adults (19–67 years old), exposed to PAHs accumulated in their households. For each age group, ILCRs for central and worst case scenario were calculated, whereby the central case scenario was based on the median mass fraction of individual PAH compounds and average dust intake rates, while the worst case scenario was based on the maximum measured mass fraction of individual PAHs obtained in analyzed house dust samples and high dust intake rates. The cancer risk levels for exposure routes of dust ingestion and dermal contact are shown in Figure 3. Total ILCR values obtained for children and adults in our study were comparable for both scenarios and were 2.18 × 10^−7^ and 2.64 × 10^−7^ for children and adults, respectively, in the central case scenario, and 1.85 × 10^−6^ and 2.28 × 10^−6^ for children and adults, respectively, in the worst case scenario. According to the US EPA, one in a million cases has the potential to develop an additional human cancer over a lifetime (ILCR = 10^−6^). This value is considered to be an acceptable level risk. According to our results, ILCR_tot_ obtained for the central case scenarios were lower than the specified limit value, but in the worst case scenario, the acceptable risk level for both age groups was slightly exceeded, which indicates a potential risk for the exposed Croatian population from PAHs accumulated in house dust samples. Dermal contact proved to be a more significant route of exposure for Croatian adults contributing with 88%, and 71% to the potential risk of cancer, as well as for children, contributing with 85%, and 70% to the potential risk of cancer, in the central and worst case scenarios, respectively. Several other authors also indicated a dermal exposure as the most dominant route of human exposure to PAHs [2,19,40] which points to the fact that more attention should be paid to the dermal route of PAH intake, especially in the case of younger children whose skin is more often in closer contact with dust. 

Results of the ILCR_tot_ obtained in this study were similar to the moderate scenario ILCRs determined for residents in Greece (1.2 × 10^−7^) and an order of magnitude higher than that of their worst case scenario (5.4 × 10^−7^) [22]. Compared to another recent study from Greece, similar total cancer risk was observed for adults (5.5 × 10^−6^), while they reported an order of magnitude higher total cancer risk for children (1.7 × 10^−5^) [10]. Few orders of magnitude higher total ILCRs for both children and adults were reported for Serbian households [20], and their results indicated that dermal contact was the primary exposure pathway for both age groups, unlike Besis et al. [10] who reported four- to six-fold higher ILCR_ing_ values than ILCR_derm_ for both age groups.

## 4. Conclusions

To the best of our knowledge, data obtained in this research provide the first data on PAH levels in indoor environments in Croatia, and mass fractions of PAHs measured in Croatian dust samples were at levels representing the lower end of mass fraction ranges reported in the residential dust samples worldwide. All analyzed PAH compounds were detected in all household dust samples, except BjF, which was detected in only one dust sample, while Flu and Pyr were the most abundant compounds. Although elevated indoor PAH concentrations are often associated with smoking, in our investigation statistically significantly (*p* < 0.05) higher concentrations in households where smoking was reported were observed only for DahA, which may be a result of a relatively small number of households that reported in-house smoking. Accordingly, DahA was the only compound that did not show statistically significantly positive correlation with other analysed PAHs, indicating that unlike other compounds, it originated from different sources. PCA analysis supported that statement. A statistically significant difference in mass fractions of the majority of PAH compounds, and for ∑PAH were observed between households that used wood for heating and households heated by the heating plants, with higher PAH mass fractions of Chry, Bbf, BkF, BaP, IP, and ∑PAHs in households that used wood for heating. Different approaches used to identify PAH sources suggested that mixed sources contributed to PAHs levels present in Croatian households. Concerning human health risk assessment, total ILCR values calculated for Croatian children and adults revealed that people exposed to the highest mass fractions of PAHs measured in this area are at elevated cancer risk. Interestingly, total cancer risk for children was dominated by dermal absorption, while dust ingestion was a more significant route of exposure for adults. Our study confirmed that PAH levels present in Croatian households are not negligible, and the obtained results contribute to the knowledge of the PAH content in indoor dust in East Europe.

## Figures and Tables

**Table 1:** Summary data of PAH mass fractions (ng g^−1^) in household dust samples from the city of Zagreb.

PAHs	Min	Max	Median	Geometric Mean	5th Percentile	95th Percentile
Flu	29.7	475.8	109.0	113.3	43.6	298.7
Pyr	19.4	362.6	101.8	95.9	42.5	253.3
BaA	3.0	81.9	18.6	18.9	5.4	58.3
Chry	1.8	248.3	23.9	19.9	2.9	102.0
BjF	0.0	88.8	12.4		1.9	53.2
BbF	0.9	161.5	35.8	31.2	10.5	99.2
BkF	2.9	70.2	12.9	12.7	4.1	36. 8
BaP	2.9	106.1	17.2	17.3	5.2	68.9
DahA	1.6	63.2	4.6	5.3	2.7	22.6
BghiP	10.3	255.4	74.2	67.4	17.2	174.7
IP	4.4	122.4	20.9	21.6	5.6	81.4
ΣPAHs	92.9	1504.1	466.8	447.4	170.5	1097.3

**Table 2:** Mass fraction ranges of PAHs in house dust samples from residential indoor environments around the world (ng g^−1^).

Measuring Place	Sampling Year	No. of Measured PAHs	ΣPAH (ng g^−1^)	Reference
Italy	2006	16	36–34,453	[18]
Canada	2002–2003	13	1500–325,000	[8]
California	2005–2007	16	163–4390	[17]
Greece	2010	16	26.2–1394	[21]
Brazil	2008	16	400–13,310	[37]
China	2012	18	2180–14,200	[9]
Czech Republic	2013	15	39.1 ± 9.43 ^a^	[23]
Saudi Arabia	2014–2015	15	950–11,950	[38]
Kuwait	2014–2015	15	450–9100	[38]
China	2014–2015	16	21,800–329,600	[36]
Nigeria	2016	16	205–2963	[14]
Saudi Arabia	/	13	55–16,275	[39]
Ecuadorian Amazonia	2017	16	130–29,200	[5]
Kuwait	2018	16	450–2242	[40]
Iran	2018	16	131–429	[41]
Greece	2017	16	703–13,200	[10]
Greece	2015	25	1400–7300	[22]
Saudi Arabia	2020	13	2310–37,665	[15]
Serbia	2021	16	140–8265	[20]
Croatia	2020–2021	11	92.9–1504.1	This study

^a^—average value; /—data not provided.

**Table 3:** Spearman correlation coefficients (r) between mass fractions of PAHs detected in household dust samples from the city of Zagreb (significant correlations (*p* < 0.001) are shown in bold).

	Pyr	BaA	Chry	BjF	BbF	BkF	BaP	DahA	BghiP	IP
Flu	**0.956**	**0.858**	**0.629**	**0.665**	**0.817**	**0.756**	**0.780**	**0.354 ^a^**	**0.604**	**0.726**
Pyr		**0.848**	**0.642**	**0.622**	**0.811**	**0.790**	**0.785**	**0.371 ^a^**	**0.648**	**0.751**
BaA			**0.580**	**0.657**	**0.778**	**0.742**	**0.743**	**0.285 ^b^**	**0.560**	**0.746**
Chry				**0.431**	**0.595**	**0.496**	**0.559**	**0.239**	**0.439**	**0.389 ^a^**
BjF					**0.748**	**0.669**	**0.687**	**0.180**	**0.486**	**0.686**
BbF						**0.905**	**0.884**	**0.322 ^a^**	**0.576**	**0.874**
BkF							**0.853**	**0.341 ^a^**	**0.589**	**0.874**
BaP								**0.383 ^a^**	**0.529**	**0.854**
DahA									**0.131**	**0.328 ^a^**
BghiP										**0.578**

^a^ Significant at the 0.01; ^b^ Significant at the 0.05.

**Table 4:** Spearman correlation coefficients (r) between PAH mass fractions measured in house dust samples from the city of Zagreb and data collected on corresponding household factors (significant correlations (*p* < 0.05) are shown in bold).

	Flu	Pyr	BaA	Chry	BjF	BbF	BkF	BaP	DahA	BghiP	IP	ΣPAHs
Age of the house	0.111	0.106	0.111	0.111	0.109	0.227	**0.245**	0.223	0.121	−0.004	0.221	0.114
Number of residents	−0.019	−0.035	−0.095	0.007	0.063	−0.079	−0.052	−0.061	0.056	−0.019	−0.099	−0.043
House area	0.089	0.094	0.045	−0.047	**0.276**	0.196	0.128	0.159	−0.117	0.143	0.197	0.138
Carpeted area	−0.014	−0.047	−0.054	0.007	**0.300**	0.126	0.069	0.180	0.021	0.112	0.120	0.085
Number of upholstered furniture	−0.017	−0.019	−0.109	−0.106	0.081	−0.006	−0.014	0.001	0.106	−0.067	0.018	−0.023
Number of windows	0.022	0.004	−0.047	−0.091	0.231	0.135	0.079	0.172	−0.020	−0.019	0.103	0.060
Balcony area	0.043	0.111	−0.040	−0.087	0.074	0.039	0.090	0.002	0.001	0.201	0.108	0.073
Number of curtains	0.165	0.157	0.185	0.062	**0.359**	0.228	**0.252**	**0.282**	0.130	**0.256**	**0.278**	0.237
Number of electronic devices	0.161	0.145	0.110	0.054	**0.298**	0.192	**0.249**	0.197	0.076	0.081	0.198	0.184
Active use of electronic devices	0.206	0.212	0.168	**0.500**	**0.254**	**0.257**	0.225	**0.267**	0.004	0.128	0.149	**0.275**

## Data Availability

The data presented in this study are available on request from the corresponding author. The data are not publicly available due to privacy.

## Supplementary Materials

The following supporting information can be downloaded at: https://www.mdpi.com/article/10.3390/ijerph191911848/s1, Table S1: Risk parameters for different age groups, Refs [46,47] are cited in Supplementary Materials.

## References

[1] Maertens R.M., Bailey J., White P.A. (2004). The Mutagenic Hazards of Settled House Dust: A Review. Mutat. Res. Rev. Mutat. Res..

[2] Al-Harbi M., Al-Enzi E., Al-Mutairi H., Whalen J.K. (2021). Human Health Risks from Brominated Flame Retardants and Polycyclic Aromatic Hydrocarbons in Indoor Dust. Chemosphere.

[3] Chakraborty P., Prithiviraj B., Selvaraj S., Kumar B. (2016). Polychlorinated Biphenyls in Settled Dust from Informal Electronic Waste Recycling Workshops and Nearby Highways in Urban Centers and Suburban Industrial Roadsides of Chennai City, India: Levels, Congener Profiles and Exposure Assessment. Sci. Total Environ..

[4] Harrad S., Abdallah M.A.E., Oluseyi T. (2016). Polybrominated Diphenyl Ethers and Polychlorinated Biphenyls in Dust from Cars, Homes, and Offices in Lagos, Nigeria. Chemosphere.

[5] Tan S.Y., Praveena S.M., Abidin E.Z., Cheema M.S. (2016). A Review of Heavy Metals in Indoor Dust and Its Human Health-Risk Implications. Rev. Environ. Health.

[6] Tao F., Sellström U., Wit C.A. (2019). De Organohalogenated Flame Retardants and Organophosphate Esters in Office Air and Dust from Sweden. Environ. Sci. Technol..

[7] Velázquez-Gómez M., Lacorte S. (2020). Organic Pollutants in Indoor Dust from Ecuadorian Amazonia Areas Affected by Oil Extractivism. Environ. Res..

[8] Maertens R.M., Yang X., Zhu J., Gagne R.W., Douglas G.R., White P.A. (2008). Mutagenic and Carcinogenic Hazards of Settled House Dust I: Polycyclic Aromatic Hydrocarbon Content and Excess Lifetime Cancer Risk from Preschool Exposure. Environ. Sci. Technol..

[9] Yang Q., Chen H., Li B. (2015). Polycyclic Aromatic Hydrocarbons (PAHs) in Indoor Dusts of Guizhou, Southwest of China: Status, Sources and Potential Human Health Risk. PLoS ONE.

[10] Besis A., Botsaropoulou E., Balla D., Voutsa D., Samara C. (2021). Toxic Organic Pollutants in Greek House Dust: Implications for Human Exposure and Health Risk. Chemosphere.

[11] (2010). WHO, IARC Monographs on the Evaluation of Carcinogenic Risks to Humans, Volume 92, Some Non-heterocyclic Polycyclic Aromatic Hydrocarbons and Some Related Exposures. https://publications.iarc.fr/Book-And-Report-Series/Iarc-Monographs-On-The-Identification-Of-Carcinogenic-Hazards-To-Humans/Some-Non-heterocyclic-Polycyclic-Aromatic-Hydrocarbons-And-Some-Related-Exposures-2010.

[12] Naspinski C., Lingenfelter R., Cizmas L., Naufal Z., He L.Y., Islamzadeh A., Li Z., Li Z., McDonald T., Donnelly K.C. (2008). A Comparison of Concentrations of Polycyclic Aromatic Compounds Detected in Dust Samples from Various Regions of the World. Environ. Int..

[13] Dubowsky S.D., Wallace L.A., Buckley T.J. (1999). The Contribution of Traffic to Atmospheric Concentrations of Polycyclic Aromatic Hydrocarbons. Environ. Sci. Technol..

[14] Iwegbue C.M.A., Obi G., Uzoekwe S.A., Egobueze F.E., Odali E.W., Tesi G.O., Nwajei G.E., Martincigh B.S. (2019). Distribution, Sources and Risk of Exposure to Polycyclic Aromatic Hydrocarbons in Indoor Dusts from Electronic Repair Workshops in Southern Nigeria. Emerg. Contam..

[15] Alamri S.H., Ali N., Albar H.M.S.A., Rashid M.I., Rajeh N., Qutub M.M.A., Malarvannan G. (2021). Polycyclic Aromatic Hydrocarbons in Indoor Dust Collected during the Covid-19 Pandemic Lockdown in Saudi Arabia: Status, Sources and Human Health Risks. Int. J. Environ. Res. Public Health.

[16] Abdel-Shafy H.I., Mansour M.S.M. (2016). A Review on Polycyclic Aromatic Hydrocarbons: Source, Environmental Impact, Effect on Human Health and Remediation. Egypt. J. Pet..

[17] Hoh E., Hunt R.N., Quintana P.J.E., Zakarian J.M., Chatfield D.A., Wittry B.C., Rodriguez E., Matt G.E. (2012). Environmental Tobacco Smoke as a Source of Polycyclic Aromatic Hydrocarbons in Settled Household Dust. Environ. Sci. Technol..

[18] Mannino M.R., Orecchio S. (2008). Polycyclic Aromatic Hydrocarbons (PAHs) in Indoor Dust Matter of Palermo (Italy) Area: Extraction, GC-MS Analysis, Distribution and Sources. Atmos. Environ..

[19] Cao Z., Wang M., Chen Q., Zhu C., Jie J., Li X., Dong X., Miao Z., Shen M., Bu Q. (2019). Spatial, Seasonal and Particle Size Dependent Variations of PAH Contamination in Indoor Dust and the Corresponding Human Health Risk. Sci. Total Environ..

[20] Živančev J., Antić I., Buljovčić M., Đurišić-Mladenović N. (2022). A Case Study on the Occurrence of Polycyclic Aromatic Hydrocarbons in Indoor Dust of Serbian Households: Distribution, Source Apportionment and Health Risk Assessment. Chemosphere.

[21] Christopoulou O.D., Sakkas V.A., Albanis T.A. (2012). Evaluation of Matrix Solid-Phase Dispersion Extraction for the Determination of Polycyclic Aromatic Hydrocarbons in Household Dust with the Aid of Experimental Design and Response Surface Methodology. J. Sep. Sci..

[22] Stamatelopoulou A., Dasopoulou M., Bairachtari K., Karavoltsos S., Sakellari K., Maggos T. (2021). Contamination and Potential Risk Assessment of Polycyclic Aromatic Hydrocarbons (PAHS) and Heavy Metals in House Settled Dust Collected from Residences of Young Children. Appl. Sci..

[23] Melymuk L., Bohlin-Nizzetto P., Vojta Š., Krátká M., Kukučka P., Audy O., Přibylová P., Klánová J. (2016). Distribution of Legacy and Emerging Semivolatile Organic Compounds in Five Indoor Matrices in a Residential Environment. Chemosphere.

[24] Klinčić D., Tariba Lovaković B., Jagić K., Dvoršćak M. (2021). Polybrominated Diphenyl Ethers and the Multi-Element Profile of House Dust in Croatia: Indoor Sources, Influencing Factors of Their Accumulation and Health Risk Assessment for Humans. Sci. Total Environ..

[25] Jakovljević I., Pehnec G., Vadjić V., Šišović A., Davila S., Bešlić I. (2015). Carcinogenic Activity of Polycyclic Aromatic Hydrocarbons Bounded on Particle Fraction. Environ. Sci. Pollut. Res..

[26] Pehnec G., Jakovljević I. (2018). Carcinogenic Potency of Airborne Polycyclic Aromatic Hydrocarbons in Relation to the Particle Fraction Size. Int. J. Environ. Res. Public Health.

[27] Ma Y., Liu A., Egodawatta P., McGree J., Goonetilleke A. (2017). Quantitative Assessment of Human Health Risk Posed by Polycyclic Aromatic Hydrocarbons in Urban Road Dust. Sci. Total Environ..

[28] Nisbet I.C.T., LaGoy P.K. (1992). Toxic Equivalency Factors (TEFs) for Polycyclic Aromatic Hydrocarbons (PAHs). Regul. Toxicol. Pharmacol..

[29] (1989). Risk Assessment Guidance for Superfund. Volume I Human Health Evaluation Manual (Part A).

[30] Qi H., Li W.L., Zhu N.Z., Ma W.L., Liu L.Y., Zhang F., Li Y.F. (2014). Concentrations and Sources of Polycyclic Aromatic Hydrocarbons in Indoor Dust in China. Sci. Total Environ..

[31] Šišović A., Pehnec G., Jakovljević I., Šilović Hujić M., Vađić V., Bešlić I. (2012). Polycyclic Aromatic Hydrocarbons at Different Crossroads in Zagreb, Croatia. Bull. Environ. Contam. Toxicol..

[32] Jakovljević I., Žužul S. (2011). Policiklički Aromatski Ugljikovodici u Zraku. Arh. Hig. Rada Toksikol..

[33] Lawal A.T. (2017). Polycyclic Aromatic Hydrocarbons. A Review. Cogent Environ. Sci..

[34] Ravindra K., Sokhi R., Van Grieken R. (2008). Atmospheric Polycyclic Aromatic Hydrocarbons: Source Attribution, Emission Factors and Regulation. Atmos. Environ..

[35] Maliszewska-Kordybach B. (1996). Polycyclic Aromatic Hydrocarbons in Agricultural Soils in Poland: Preliminary Proposals for Criteria to Evaluate the Level of Soil Contamination. Appl. Geochem..

[36] Wang Z., Wang S., Nie J., Wang Y., Liu Y. (2017). Assessment of Polycyclic Aromatic Hydrocarbons in Indoor Dust from Varying Categories of Rooms in Changchun City, Northeast China. Environ. Geochem. Health.

[37] Coronas M.V., Bavaresco J., Rocha J.A.V., Geller A.M., Caramão E.B., Rodrigues M.L.K., Vargas V.M.F. (2013). Attic Dust Assessment near a Wood Treatment Plant: Past Air Pollution and Potential Exposure. Ecotoxicol. Environ. Saf..

[38] Ali N., Ismail I.M.I., Khoder M., Shamy M., Alghamdi M., Costa M., Ali L.N., Wang W., Eqani S.A.M.A.S. (2016). Polycyclic Aromatic Hydrocarbons (PAHs) in Indoor Dust Samples from Cities of Jeddah and Kuwait: Levels, Sources and Non-Dietary Human Exposure. Sci. Total Environ..

[39] Ali N. (2019). Polycyclic Aromatic Hydrocarbons (PAHs) in Indoor Air and Dust Samples of Different Saudi Microenvironments; Health and Carcinogenic Risk Assessment for the General Population. Sci. Total Environ..

[40] Al-Harbi M., Alhajri I., Whalen J.K. (2020). Health Risks Associated with the Polycyclic Aromatic Hydrocarbons in Indoor Dust Collected from Houses in Kuwait. Environ. Pollut..

[41] Nazmara S., Sorooshian A., Delikhoon M., Baghani A.N., Ashournejad Q., Barkhordari A., Basmehchi N., Kasraee M. (2020). Characteristics and Health Risk Assessment of Polycyclic Aromatic Hydrocarbons Associated with Dust in Household Evaporative Coolers. Environ. Pollut..

[42] Agudelo-Castañeda D.M., Teixeira E.C. (2014). Seasonal Changes, Identification and Source Apportionment of PAH in PM_1.0_. Atmos. Environ..

[43] Chang K.F., Fang G.C., Chen J.C., Wu Y.S. (2006). Atmospheric Polycyclic Aromatic Hydrocarbons (PAHs) in Asia: A Review from 1999 to 2004. Environ. Pollut..

[44] Teixeira E.C., Agudelo-Castañeda D.M., Fachel J.M.G., Leal K.A., Garcia K.d.O., Wiegand F. (2012). Source Identification and Seasonal Variation of Polycyclic Aromatic Hydrocarbons Associated with Atmospheric Fine and Coarse Particles in the Metropolitan Area of Porto Alegre, RS, Brazil. Atmos. Res..

[45] Ren Y., Cheng T., Chen J. (2006). Polycyclic Aromatic Hydrocarbons in Dust from Computers: One Possible Indoor Source of Human Exposure. Atmos. Environ..

[46] (2011). Exposure Factors Handbook: 2011 Edition.

[47] (2017). Exposure Factors Handbook Chapter 5 (Update): Soil and Dust Ingestion.

