# Supplementary material for: Polycyclic Aromatic Hydrocarbons in Indoor Dust in Croatia: Levels, Sources, and Human Health Risks
Source: Int J Environ Res Public Health. 2022 Sep 20;19(19):11848. doi: 10.3390/ijerph191911848 (PMC9565587; doi:10.3390/ijerph191911848)
Supplement: Supplementary file 1 [file ijerph-19-11848-s001.zip › ijerph-1886354-supplementary.pdf]

Table S1. Risk parameters for different age groups.

**Variable definition**	**Unit**	**Children**	**Adult**	**References**
Body weight (BW)	kg	15	70	[46]
Exposure frequency (EF)	day year^{-1}	350	350	[40]
Exposure duration (ED)	year	3	64	[40]
Ingestion rate; average/high (IR_{ing})	mg day^{-1}	40/100	20/60	[47]
Dermal exposure area (SA)	cm^{2}	2800	5700	[46]
Dermal adherence factor (AF)	mg cm^{-2}	0.2	0.07	[40]
Dermal adsorption fraction (ABS)	Unitless	0.13	0.13	[40]
Averaging life span (AT)	day	25550	25550	[22]
Ingestion carcinogenic slope (CSF_{Ing}) factor	(mg kg^{-1} day^{-1})$^{-1}$	7.3	7.3	[22]
Dermal carcinogenic slope (CSF_{Derm}) factor	(mg kg^{-1} day^{-1})$^{-1}$	25	7.3	[40]
